# Supplementary material for: Surgical treatment of spondylolisthesis by oblique lumbar interbody fusion and transpedicular screw fixation: Comparison between conventional double position versus navigation-assisted single lateral position
Source: PLoS One. 2023 Sep 14;18(9):e0291114. doi: 10.1371/journal.pone.0291114 (PMC10501584; doi:10.1371/journal.pone.0291114)
Supplement: Attachment — Submitted filename: PLOS_ONE_Response_to_Reviewers.docx [file pone.0291114.s003.docx]

Dear Dr. Kim,

Thank you for submitting your manuscript to PLOS ONE. After careful consideration, we feel that it has merit but does not fully meet PLOS ONE’s publication criteria as it currently stands. Therefore, we invite you to submit a revised version of the manuscript that addresses the points raised during the review process.

Response: We appreciate your thorough review of our manuscript. Below, you will find point-by-point responses to each comment provided by the reviewers.

General:

The title of the manuscript is not representing the core principle of the methodology (spinal navigation) and findings of the manuscript

Response: We appreciate your comment on our manuscript. We agree that our title requires correction. “Efficacy of navigation-guided pedicle screw fixation followed by oblique lumbar interbody fusion for the reduction of spondylolisthesis” would be a more appropriate title.

Lines 28-30 & 61-63: Oblique lumbar interbody fusion (OLIF) procedures are divided into anterior insertion of interbody cage first in lateral position and insertion of pedicle screw and rod system in a prone position (OLIF-con). I absolutely disagree with the authors. OLIF is an anterior interbody fusion performed through lateral approach involves an oblique channel between the prevertebral venous structures and the psoas muscle. Transpedicular fixation is not a part of OLIF technique. It is a supplementary ‘complementary” fixation that adds to the stability of the construct. This point is very important, makes the manuscript confusing at several sentences and should be corrected.

Response: We fully agree that the term “OLIF” should be specifically limited to anterior interbody fusion performed through a lateral approach, and it does not inherently include pedicle screw fixation (PSF). We routinely included PSF in the OLIF surgical procedure because of the high incidence of subsidence reported when OLIF was not reinforced with PSF. Choi et al. demonstrated a relatively higher incidence of cage subsidence in the South Korean population (Choi et al., “Subsidence after anterior lumbar interbody fusion using paired stand-alone rectangular cages”, European Spine Journal, 2006). In light of these findings, we have implemented a routine practice of inserting pedicle screws when performing OLIF procedures.

Furthermore, previous studies such as Zhao et al., “Clinical and radiological evaluation of cage subsidence following oblique lumbar interbody fusion combined with anterolateral fixation” (BMC Musculoskeletal Disorders, 2022), and Calvo-Echenique et al., “Is there any advantage of using stand-alone cages? A numerical approach” (BioMedical Engineering OnLine, 2019), also had specifically addressed the issue of cage subsidence after anterior lumbar interbody fusion. To clarify the meaning of OLIF, OLIF-con, and OLIF-one, we have made modifications to the relevant sentences.

Lines 30-38;

Oblique lumbar interbody fusion (OLIF) procedures involve anterior insertion of interbody cage in lateral position. Following OLIF, insertion of pedicle screws and rod system is performed in a prone position (OLIF-con). The location of the cage is important for restoration of lumbar lordosis and indirect decompression. However, inserting the cage at the desired location is difficult without reduction of spondylolisthesis, and reduction after insertion of interbody cage may limit the amount of reduction. Recent introduction of spinal navigation enabled both surgical procedures in one lateral position (OLIF-one). Therefore, reduction of spondylolisthesis can be performed prior to insertion of interbody cage.

Lines 73-82;

The recent introduction of spinal navigation has enabled anterior and posterior surgical procedures to be performed in one lateral position (OLIF-one) [6,7]. Consequently, the surgeon can move to either the anterior or posterior aspect of the patient, as both approaches can be used at the same time, allowing for reduction of spondylolisthesis prior to insertion of interbody cage. During OLIF-one, percutaneous pedicle screw fixation is first performed under the guidance of spinal navigation from the back. Anteriorly, the tight annulus and disc space are released during the removal of disc and restoration of disc height by serially inserting trial cages.

Introduction

Lines 59-60: “For these purposes, an interbody cage is usually inserted into the anterior one-third of the disc space in OLIF [5]”. The same meaning was repeated again in lines 63-64: “The location of the cage is crucial for restoring lumbar lordosis and providing indirect decompression” This repetition should be omitted.

Response: Thank you for commenting the redundancy. We have removed the sentence “The location of the cage is crucial for restoring lumbar lordosis and providing indirect decompression” in lines 67-68 (according to track changes version) to avoid duplication.

Lines 70-72: “Consequently, the surgeon can switch the sequences of posterior and anterior surgical procedures in one position by moving the front and back of the patient” This sentence is unclear and confusing. I think it should be corrected to: “The surgeon can move to anterior or posterior aspect of the patient because both approaches could be used at the same time.”

Response: Thank you for the correction. We have revised the sentence in lines 74-78 (according to track changes version) as follows.

Lines 74-78;

Consequently, the surgeon can move to either the anterior or posterior aspect of the patient, as both approaches can be used at the same time, allowing for reduction of spondylolisthesis prior to insertion of interbody cage.

Line 76: I think the term “inserted” should be better used instead of “located”

Response: The recommended word is more appropriate. We have revised the word “located” to “inserted” in line 84 (according to track changes version).

Methods

Lines 88-91: “All patients with spondylolisthesis greater than 3mm were included without any exclusion criteria, as the purpose of the study was to compare immediate postoperative radiologic and clinical outcomes” What is the relation between inclusion of all patients with spondylolisthesis greater than 3 mm and the purpose to study immediate post-operative radiologic and clinical outcomes?

Response: We understand that the sentences in lines 99-101 (according to track changes version) may have caused some confusion. We included patients with more than 3 mm anterior slip based on a prospective study that showed similar outcomes between decompression surgery and fusion surgery when the slip was less than 3 mm (Austevoll et al., “Decompression with or without Fusion in Degenerative Lumbar Spondylolisthesis”, The New England Journal of Medicine, 2021).

The goals of fusion surgery for spondylolisthesis are decompression, re-alignment of anterior vertebral slip, and correction of instability. If direct decompression and fusion surgery are performed, re-alignment of spondylolisthesis may not be a significant concern. However, in OLIF, which relies on indirect decompression as the core technique, re-alignment is necessary for effective indirect decompression. Therefore, we analyzed the effectiveness of reduction of spondylolisthesis as part of the initial surgical procedure. The sentence in line 99-101 (according to track changes version) has been deleted, and the rationale for inclusion criteria has been added in the discussion in lines 259-269 (according to track changes version).

Lines 94-98;

The study included 72 consecutive patients who underwent surgery [9-11] from January 2017 to August 2022 at two different institutes [Institution 1 and Institution 2; blinded for review] and had spondylolisthesis grade I according to Meyerding’s classification with slippage greater than 3mm.

Lines 259-269;

We included patients with more than 3mm of anterior slip based on the findings of a prospective study that showed similar outcomes between decompression surgery and fusion surgery when the slip was less than 3mm [12,13,15]. The goals of fusion surgery for spondylolisthesis involve decompression, re-alignment of anterior vertebral slip, and correction of instability. While re-alignment of spondylolisthesis may not be a significant concern in cases of direct decompression and fusion surgery [2], it is crucial for effective indirect decompression, which is the core technique of OLIF [14,15]. In this regard, we analyzed the effectiveness of reduction of spondylolisthesis as part of the initial surgical procedure (OLIF-one).

Lines 140-141: “Then, the surgeon moved to the back of the patient, and used the same specialized reducing device as in OLIF-con, with an undersized trial cage, to reduce the spondylolisthesis.” This sentence is confusing. The surgeons have already inserted the trial cage through the lateral position then moved to the back of the patient to do reduction utilizing the reduction system. So, I think the words “with an undersized trial cage” should be deleted.

Response: We have deleted “with an undersized trial cage” and changed the sentence in lines 153-154 (according to track changes version) as follows.

Lines 153-154;

Then, the surgeon moved to the back of the patient, and used the same specialized reducing device as in OLIF-con to reduce the spondylolisthesis.

Lines 142- 143: “After the reduction of spondylolisthesis, the set screws were inserted but not broken off to allow for sliding of the vertebra along the rod during the insertion of the interbody cage.” This sentence is confusing and unclear.

Response: We understand that the sentence in lines 155-157 (according to track changes version) may have caused confusion. We have revised the sentence as follows.

Lines 155-157;

The set screws were inserted but not fully tightened to allow for the sliding of the head of the pedicle screws along the rod during the reduction of spondylolisthesis and insertion of the interbody cage.

Results

Lines 190-192: “Surgical time was not significantly different between OLIF-con (268 ± 82.5 minutes) and OLIF-one (215 ± 42.8 minutes) (p = .10).” Better to be changed to “Surgical time was shorter in OLIF-one (215 ± 42.8 minutes) than in OLIF-con (268 ± 82.5 minutes), however the difference did not reach statistical significance (p = .10).”

Response: We have revised the sentence in lines 205-208 (according to track changes version) as follows.

Lines 205-208;

Surgical time in OLIF-one (215 ± 42.8 minutes) was shorter than that in OLIF-con (268 ± 82.5 minutes), but the difference did not reach statistical significance (p = .10).

Table 1:

The statistical difference between the 2 groups as regards male/female ratio should be reported. I think this can be examined by 2x2 contiguency table. The same as regards the difference between the number of levels in the 2 groups utilizing MxN contiguncy tables.

Response: The statistical differences between the two groups were analyzed using contingency tables.

**Table 1. Patient demographics.**

|  | OLIF-con | OLIF-one | p-value |
| --- | --- | --- | --- |
| Number of patients | 42 | 30 |  |
| Age (years) | 66.5±7.4 | 67.6±7.9 | 0.92 |
| Sex (Male : Female) | 32 : 10 | 23 : 7 | **0.96** |
| BMI (kg/m^2^) | 25.6±3.1 | 24.2±3.1 | 0.21 |
| Surgery level(s) |  |  | **0.14** |
| One | 28 | 25 |  |
| Two | 10 | 5 |  |
| Three | 4 | 0 |  |
| Level of spondylolisthesis |  |  | **0.63** |
| L2-3 | 5 | 6 |  |
| L3-4 | 8 | 3 |  |
| L4-5 | 20 | 15 |  |
| L5-S1 | 9 | 6 |  |
| Preoperative NRS-B (/10) | 7.2±2.4 | 7±2.2 | 0.76 |
| Preoperative NRS-L (/10) | 7.2±3.0 | 7.7±1.9 | 0.53 |
| Preoperative ODI (%) | 53.1±17.7 | 48.4±26.3 | 0.42 |

Conclusion

Lines 300-302: “This is the first study to demonstrate the outcome of switching the procedures during OLIF using surgical navigation. OLIF-one facilitated the reduction of spondylolisthesis and precise cage placement at the optimal position.” This sentence should be corrected to: “This is the first study to show superior results of performing OLIF with supplementary transpedicular fixation of spondylolisthesis in one lateral position utilizing spinal navigation system. Even more, this technique facilitated the reduction of spondylolisthesis and precise cage placement at the optimal position”

Response: We have revised the sentences in lines 325-329 (according to track changes version) as follows.

Lines 325-329;

This is the first study to demonstrate the superior outcomes of performing OLIF with supplementary pedicle screw fixation for patients with spondylolisthesis using a spinal navigation system in one lateral position. Moreover, this technique facilitated the reduction of spondylolisthesis and precise cage placement at the optimal position.

Review Comments to the Author

Reviewer #1:

1. Spondylolisthesis are heterogenous group based on etiology, disc height and degree of severity, please include those information as well.

Response: We appreciate the comment. We acknowledge that patients with spondylolisthesis encompass a heterogeneous group. All patients included had Meyerding’s grade I spondylolisthesis, and slippage was more than 3mm. Our study was designed as a preliminary investigation of performing OLIF with supplementary pedicle screw fixation using a spinal navigation system, with a primary focus on the reduction of spondylolisthesis and cage placement. Because the main purpose of this study was to compare the amount of reduction of spondylolisthesis, we did not measure disc height. We are currently conducting further research on OLIF-one, and these aspects will be thoroughly addressed in our subsequent studies.

Lines 94-98;

The study included 72 consecutive patients who underwent surgery [9-11] from January 2017 to August 2022 at two different institutes [Institution 1 and Institution 2; blinded for review] and had spondylolisthesis grade I according to Meyerding’s classification with slippage greater than 3mm.

2. Please give information about detailed information about the temporary sensory and motor deficit.

Response: Transient sensory deficits were characterized by hypesthesia, while transient motor deficits manifested as weakness in the corresponding nerve of the surgical level.

Lines 168-175;

The following data were reviewed: patients’ demographic data (age, sex, body mass index [BMI, kg/m2], and surgical level), intraoperative data (anesthesia time, surgical time from skin to skin, and injury to vessel, peritoneum, or bowel), complications (postoperative motor deficit (weakness on leg), temporary sensory changes (hypesthesia), surgical site infection, ileus, or hematoma), and radiological outcomes (preoperative spondylolisthesis slippage [pre-slip, Fig 2A], postoperative spondylolisthesis slippage [post-slip, Fig 2B], reduction degree of spondylolisthesis [1- post-slip/pre-slip], and the location of the cage [location % from anterior margin of caudal vertebra to the center of cage, Fig 3]).

Lines 180-181;

Complications such as temporary motor and sensory deficit were also compared.

Reviewer #2:

• Line 78: Term OLIF con used. This terminology probably explained in line 73. Kindly do it clearly. Similarly, it is confusing sequence at line 118.

Response: To clarify the meaning of OLIF, OLIF-con, and OLIF-one, we have modified the sentences.

Lines 30-38;

Oblique lumbar interbody fusion (OLIF) procedures involve anterior insertion of interbody cage in lateral position. Following OLIF, insertion of pedicle screws and rod system is performed in a prone position (OLIF-con). The location of the cage is important for restoration of lumbar lordosis and indirect decompression. However, inserting the cage at the desired location is difficult without reduction of spondylolisthesis, and reduction after insertion of interbody cage may limit the amount of reduction. Recent introduction of spinal navigation enabled both surgical procedures in one lateral position (OLIF-one). Therefore, reduction of spondylolisthesis can be performed prior to insertion of interbody cage.

Lines 73-82;

The recent introduction of spinal navigation has enabled anterior and posterior surgical procedures to be performed in one lateral position (OLIF-one) [6,7]. Consequently, the surgeon can move to either the anterior or posterior aspect of the patient, as both approaches can be used at the same time, allowing for reduction of spondylolisthesis prior to insertion of interbody cage. During OLIF-one, percutaneous pedicle screw fixation is first performed under the guidance of spinal navigation from the back. Anteriorly, the tight annulus and disc space are released during the removal of disc and restoration of disc height by serially inserting trial cages.

Lines 131-135;

The key distinction between OLIF-one and OLIF-con was the sequence of the surgical procedures. In OLIF-con, the sequence was anterior followed by posterior. However, in OLIF-one, the surgical procedures were performed in the sequence of posterior, anterior, posterior, anterior and then posterior again.

• OLIF con is open procedure with procedure with front back sequence and OLIF one is MIS navigation procedure with back, front back sequence?

Response: We appreciate the comment. Both OLIF-con and OLIF-one used percutaneous pedicle screw fixation. The key distinction between OLIF-one and OLIF-con was the sequence of the surgical procedures. In OLIF-con, the sequence was anterior followed by posterior. However, in OLIF-one, the surgical procedures were performed in the sequence of posterior, anterior, posterior, anterior and then posterior again. We are sorry for the confusion.
