# Supplementary material for: Surgical treatment of spondylolisthesis by oblique lumbar interbody fusion and transpedicular screw fixation: Comparison between conventional double position versus navigation-assisted single lateral position
Source: PLoS One. 2023 Sep 14;18(9):e0291114. doi: 10.1371/journal.pone.0291114 (PMC10501584; doi:10.1371/journal.pone.0291114)
Supplement: Attachment — Submitted filename: PLOS_ONE_Response_to_Reviewers_minor_revision.docx [file pone.0291114.s004.docx]

Dear Dr. Chi Heon Kim,

Thank you for submitting your manuscript to PLOS ONE. After careful consideration, we feel that it has merit but does not fully meet PLOS ONE’s publication criteria as it currently stands. Therefore, we invite you to submit a revised version of the manuscript that addresses the points raised during the review process.

Response: We appreciate your thorough review of our manuscript. Below, you will find point-by-point responses to each comment provided by the reviewers.

Thank you for addressing the reviewer and editorial comments. However, in my opinion, there is still improvement potentials.

Additional Editor Comments:

The authors addressed almost all the reviewers and editorial comments. However, as regards the title, I think still the title is not representing the core importance of the study. I would suggest the following title: SURGICAL TREATMENT OF SPONDYLOLITHESIS BY OLIF AND TRANSPEDICULAR SCREW FIXATION: COMPARISNON BETWEEN CONVENTIONAL DOUBLE POSITION VERSUS NAVIGATION- ASSISTED SINGLE LATERAL POSITION. Consequently, the short title should be also changed. I would suggest: single versus double position OLIF and transpedicular screw fixation

Response: We appreciate your comment on our manuscript title. We agree that our title requires correction. “Surgical treatment of spondylolisthesis by oblique lumbar interbody fusion and transpedicular screw fixation: comparison between conventional double position versus navigation-assisted single lateral position” would be a more appropriate title. Furthermore, short title was revised as “Single versus double position OLIF and transpedicular screw fixation”.
